# High Betaine and Dynamic Increase of Betaine Levels Are Both Associated With Poor Prognosis of Patients With Pulmonary Hypertension

**DOI:** 10.3389/fcvm.2022.852009

**Published:** 2022-03-30

**Authors:** Yicheng Yang, Jing Xu, Jingjing Zhou, Jing Xue, Jianing Gao, Xin Li, Bo Sun, Beilan Yang, Zhihong Liu, Zhihui Zhao, Qin Luo, Qixian Zeng, Lemin Zheng, Changming Xiong

**Affiliations:** ^1^Center of Pulmonary Vascular Disease, State Key Laboratory of Cardiovascular Disease, Fuwai Hospital, National Center for Cardiovascular Disease, Chinese Academy of Medical Sciences and Peking Union Medical College, Beijing, China; ^2^Department of Cardiology, State Key Laboratory of Cardiovascular Disease, Fuwai Hospital, National Center for Cardiovascular Diseases, Chinese Academy of Medical Sciences and Peking Union Medical College, Beijing, China; ^3^China National Clinical Research Center for Neurological Diseases, Tiantan Hospital, Advanced Innovation Center for Human Brain Protection, The Capital Medical University, Beijing, China; ^4^The Institute of Cardiovascular Sciences and Institute of Systems Biomedicine, School of Basic Medical Sciences, Key Laboratory of Molecular Cardiovascular Sciences of Ministry of Education, NHC Key Laboratory of Cardiovascular Molecular Biology and Regulatory Peptides, Health Science Center, Peking University, Beijing, China; ^5^Department of Information Center, Fuwai Hospital, National Center for Cardiovascular Diseases, Chinese Academy of Medical Sciences and Peking Union Medical College, Beijing, China

**Keywords:** metabolites, betaine, pulmonary hypertension, severity, prognosis, Δbetaine

## Abstract

**Background and Objective:**

The association between plasma betaine levels and cardiovascular diseases (CVDs) has been revealed except for pulmonary hypertension (PH). In this study, we aimed to explore the role of betaine in patients with PH.

**Methods:**

Inpatients with PH at Fuwai Hospital were enrolled after excluding relative comorbidities. Each patient received at least one follow-up through a clinical visit, and the fasting blood was obtained both at the first and second hospitalization for betaine detection. The primary endpoint was defined as composite outcome events and the mean duration was 14.3 (6.9, 21.3) months. The associations of betaine and changes of betaine (Δbetaine) with disease severity and prognosis were explored.

**Results:**

Finally, a total of 216 patients with PH were included and the medians for betaine plasma levels in the total patients group, low betaine, and high betaine groups were 49.8 (39.0, 68.3) μM, 39.0 (33.5, 44.7) μM, and 68.1 (57.8, 88.7) μM, respectively. High betaine was associated with poor World Health Organization Functional Class (WHO-FC), increased N-terminal pro-brain natriuretic peptide (NT-proBNP), low tricuspid annular plane systolic excursion (TAPSE), and cardiac output index even after adjusting for confounders. Patients with high betaine were over twice the risk to receive the poor prognosis than those with a low level [hazard ratio (HR) = 2.080, (95% CI: 1.033–4.188)]. Moreover, the decrease of betaine level after further treatment was positively correlated to ΔNT-proBNP indicating Δbetaine might be an effector of disease severity, and dynamic increase of betaine was also associated with poor prognosis in PH.

**Conclusion:**

Betaine was associated with disease severity and might be an effector in PH. Patients with increased levels or with dynamic rise of betaine heralded a poor prognosis.

## Introduction

Pulmonary hypertension (PH), classified into five categories according to different etiology, is a kind of progressive cardiovascular disease (CVD) that results in heart failure and death eventually (1). Although the knowledge on PH pathogenesis and treatment has improved in the past decade, it is still regarded as an extremely complex disease that required comprehensive and time-consuming inspections and evaluations, and the survival of the patient is still worrying. Nowadays, how to better manage patients to improve their prognosis is a tremendous challenge.

Facing this plateau, the exploration of serological biomarkers in PH is a promising way for effective disease management (1). However, the well-recognized serological biomarker is lacking except for brain natriuretic peptide (BNP) and N-terminal pro-BNP (NT-proBNP) up to date. A growing body of literature recently implicates metabolites that include betaine, choline, and trimethylamine N-oxide in CVD risk (2–6). Betaine was obtained from food sources, such as grain products, vegetables, red meat, eggs, and fish (7), or synthesized *de novo* from the irreversible oxidation of choline *via* betaine aldehyde dehydrogenase and choline dehydrogenase predominantly in the liver, a process under homeostatic control in healthy humans (8). It has been suggested that high betaine levels may exert the prognostic value of major adverse cardiovascular event risk in table cardiac subjects who underwent elective diagnostic coronary angiography (9) and high dietary betaine intake was associated with an increased risk of incident coronary heart disease (10). Similar results were also reported in patients with diabetes mellitus (11).

To our best knowledge, the role of betaine in PH has never been investigated. Here, we aimed to investigate the association of plasma betaine concentration and disease severity of patients with PH and to examine whether betaine could be served as a biomarker in clinical outcomes. Moreover, we also preliminarily explored the value of dynamic changes of betaine (Δbetaine) in PH.

## Materials and Methods

This is a clinical study designed to evaluate the association between betaine levels and PH. The study was approved by the Ethics Committees of Fuwai Hospital and adhered to the Declaration of Helsinki. All patients were provided written informed consent.

### Study Population and Clinical Data Collection

Pulmonary hypertension was diagnosed as mean pulmonary atrial pressure (mPAP) ≥25 mmHg by right heart catheterization (RHC) in this study. PH inpatients at Fuwai Hospital Pulmonary Vascular Word in China from March 2019 to April 2020 were enrolled. Exclusion criteria included (1) patients who were diagnosed as connective tissue disease-related PH or had the immune disease; (2) patients with acute coronary syndromes, active infection, malignancy, congestive heart failure, and diabetes; (3) incomplete clinical data; and (4) patients without rehospitalization. Clinical data that include demographic characteristics, World Health Organization Functional Class (WHO-FC), laboratory parameters, echocardiography, exercise capacity, and hemodynamics were collected in this study.

### Follow-Up and Study Endpoint

Each patient received at least one follow-up through hospitalization visit and the fasting blood was obtained both at the first and second hospitalization for betaine and other clinical indicators detection. When exploring the association between betaine levels at baseline and clinical outcome, the follow-up duration was defined as the time from the first hospitalization to the occurrence of outcome or the end of follow-up. The mean duration was 14.3 (6.9, 21.3) months. In the analysis of Δbetaine, the follow-up duration started from the second hospitalization and the mean duration was 9.9 (2.7, 15.3) months.

The study endpoint was defined as composite outcome events that include death, rehospitalization due to heart failure, escalation of targeted medication due to the disease condition, deterioration of PH that includes worsening symptoms, higher WHO-FC compared with baseline, or at least 15% decreased 6-min walk distance (6MWD) from baseline (12).

### Quantification of Betaine

Collecting 5 ml of blood samples at fasting and centrifuging for 10 min at a speed of 3,000 rpm. The supernatants were obtained and stored at −80°C. After thawing, 20 μl supernatants were aliquoted to a 1.5-ml tube and mixed with 80 μl of 5 μM internal standard composed of d9-metabolites in methanol. Protein in the samples was precipitated by vortexing for 1 min. Next, the samples were centrifuged at 20,000 *g* at 4°C for 10 min. In order to obtain the precise concentration of the analytes, a standard curve was performed using 20 μl of various concentration standards (0–100 μM) processed in parallel. The coefficient of determination (R^2^) reached 0.99 in standard curves were acceptable. Supernatants (70 μl) were analyzed by injecting them onto a silica column using an LC-20AD Shimadzu pump system at a flow rate of 0.5 ml/min, SIL-20AXR Autosampler interfaced with an API 5500Q-TRAP mass spectrometer. A discontinuous gradient was generated to resolve the analytes by mixing solvent A (0.1% propanoic acid in water) with solvent B (0.1% acetic acid in methanol) at different ratios. Analytes were monitored using electrospray ionization in positive-ion mode with multiple reactions monitoring of precursor. Three quality-control samples with different betaine concentrations were measured every 20 samples.

### Statistical Analysis

A restricted cubic spline was used to explore the linear or non-linear relationship between betaine and clinical outcome. Student’s *t*-test or Wilcoxon rank sum test for continuous variables and χ^2^ test for categorical variables were used to examine the difference between groups. Paired-samples *t*-tests or paired Wilcoxon rank sum test was used to compare the changes between first and second hospitalization. Spearman’s correlation (2-tailed), univariate or multivariate logistics were used to determine correlations between betaine and clinical markers of disease severity. Spearman’s correlation (2-tailed) was also utilized for exploring the relation between Δbetaine and changes in the clinical indicator. Kaplan–Meier (KM) analysis and Cox proportional hazards regression were used for determining hazard ratios (HRs) and 95% confidence intervals (CIs). A two-sided *p* < 0.05 was considered statistically significant. Analyses performed in this study used R 2.8.0 (Vienna, Austria), SPSS (version 23; IBM Corp.) and GraphPad (GraphPad Software, Inc).

## Results

### Population Characteristics at Baseline

Finally, a total of 216 patients with PH, 140 pulmonary arterial hypertension, 61 chronic thromboembolic PH, 12 PH with multifactorial mechanisms, and 3 PH due to hypoxia, were included in this study. During the follow-up duration, 159 patients survived without clinical worsening, while 12 patients were rehospitalized for progression of PH or heart failure, 16 patients for escalation of targeted medication, and 5 patients were died.

**FIGURE 1:**
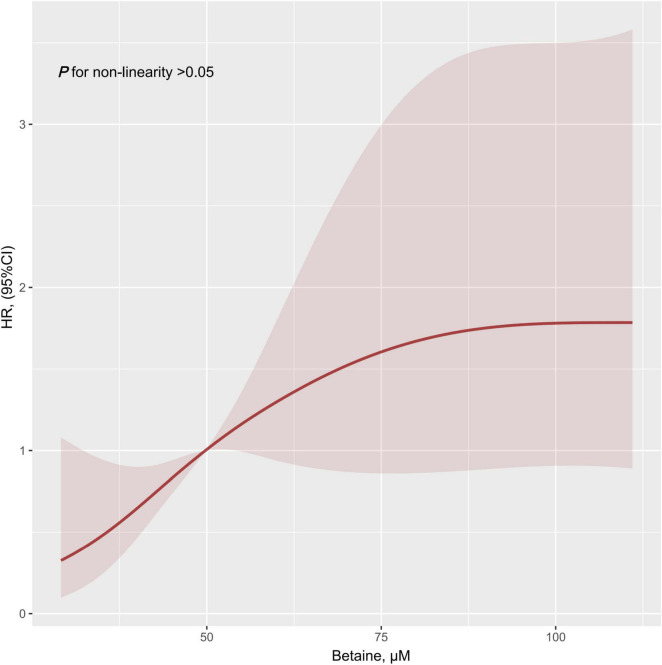
Restricted cubic spline result of plasma betaine levels in relation to hazard ratio for the risk of clinical outcomes. Dark red line with 95% CI shaded in light red. HR, hazard ratio; CI, confidence interval.

The result of the restricted cubic spline showed the linear relationship of betaine in this study (non-linear, *p* = 0.100, Figure 1). Patients with PH were stratified into low betaine and high betaine groups by 50th percentile of betaine and the basic characteristics are shown in Table 1. The medians (interquartile ranges) for betaine plasma levels in the total patients group, low betaine, and high betaine groups were 49.8 (39.0, 68.3) μM, 39.0 (33.5, 44.7) μM, and 68.1 (57.8, 88.7) μM, respectively. Patients in the high betaine group were elder and had a higher proportion of men than the low betaine group. Moreover, patients with high betaine received worse WHO-FC, higher NT-proBNP, larger right ventricular diameter (RVD), lower tricuspid annular plane systolic excursion (TAPSE), and cardiac output index than those with low betaine plasma levels (Supplementary Figure 1).

**TABLE 1:** Characteristics of patients stratified by 50th percentile of betaine.

Variables	Total patients (N = 216)	Low betaine (N = 108)	High betaine (N = 108)	*p* value
Age, years	36 (27, 54)	32 (25, 46)	43 (30, 56)	0.001
Female sex, n (%)	141 (65.3)	79 (73.1)	62 (57.4)	0.022
BMI, kg/m^2^	22.0 ± 3.9	21.5 ± 4.2	22.6 ± 3.4	0.067
**WHO-FC, n (%)**				
I–II	132 (61.1)	76 (70.4)	56 (51.9)	0.008
III–IV	84 (38.9)	32 (29.6)	52 (48.1)	0.008
**Laboratories**				
Betaine, μM	49.8 (39.0, 68.3)	39.0 (33.5, 44.7)	68.1 (57.8, 88.7)	<0.001
NT-proBNP, pg/ml	446.3 (144.3, 1563.0)	304.0 (127.0, 659.8)	821.6 (194.5, 2328.5)	<0.001
Albumin, g	42.3 ± 4.8	42.8 ± 4.5	41.8 ± 5.1	0.203
Triglycerides	1.1 (0.8, 1.5)	1.1 (0.8, 1.5)	1.1 (0.8, 1.5)	0.687
Total cholesterol, mM	4.2 ± 1.1	4.2 ± 1.1	4.1 ± 1.1	0.975
Creatinine, μM	75.0 (65.5, 90.0)	71.0 (62.4, 81.9)	82.3 (71.0, 95.0)	<0.001
**Echocardiography**				
LVEF, %	65.0 (60.0, 70.0)	65.0 (60.0, 69.3)	65.0 (60.0, 70.0)	0.533
RVD, mm	32.0 (27.0, 37.0)	30.0 (26.8, 36.0)	33.0 (27.5, 38.0)	0.04
TAPSE, mm	16.5 (14.0, 18.0)	18.0 (15.0, 19.0)	15.0 (13.0, 18.0)	0.005
**Exercise capacity**				
PeakVO_2_, mL/min/kg	47.4 ± 15.3	15.0 ± 4.1	14.1 ± 3.7	0.249
VO_2_%	1.5 ± 0.4	1.5 ± 0.4	1.4 ± 0.3	0.045
6MWD, m	422.3 ± 96.5	426.4 ± 86.8	417.3 ± 107.5	0.096
**Hemodynamics**				
mRAP, mmHg	6.0 (4.0, 8.3)	6.0 (3.5, 8.5)	7.0 (4.0, 8.5)	0.538
mPAP, mmHg	57.0 (47.0, 70.0)	57.5 (46.8, 70.5)	56.5 (47.0, 70.8)	0.726
Cardiac output index, L/min*m^2^	3.2 ± 1.0	3.4 ± 1.1	2.9 ± 0.9	0.002
PAWP, mmHg	8.0 (6.0, 11.0)	8.0 (6.0, 11.0)	9.0 (6.0, 11.0)	0.452
PVR, WU	6.7 (5.0, 11.2)	6.8 (5.0, 10.0)	6.6 (4.9, 11.7)	0.861

*Betaine represents plasma betaine concentrations and patients were stratified into low betaine and high betaine groups by 50th percentile of betaine (49.8 μM). BMI, body mass index; WHO-FC, World Health Organization Function Class; NT-proBNP, N-terminal pro-brain natriuretic peptide; LVEF, left ventricular ejection fraction; RVD, right ventricular diameter; TAPSE, tricuspid annular plane systolic excursion; 6MWD, 6-min walk distance; mRAP, mean right atrial pressure; mPAP, mean pulmonary atrial pressure; PAWP, pulmonary arterial wedge pressure; PVR, pulmonary vascular resistance.*

### Correlation Between Betaine and Disease Severity

Supplementary Table 1 shows the correlations between betaine and clinical indicators. Following adjustments for confounders that include age, sex, body mass index (BMI; Supplementary Table 2), increased betaine was also associated with WHO-FC [odds ratio (OR) = 2.349, (95% CI: 1.241–4.448), *p* < 0.009], NT-proBNP [OR = 1.993, (95% CI: 1.026–3.870), *p* = 0.042], TAPSE [OR = 2.026, (95% CI: 1.087–3.779), *p* = 0.026], and cardiac output index [OR = 2.390 (95% CI: 1.087–5.255), *p* = 0.030].

### High Betaine Was Associated With Poor Prognosis of Patients With PH

**FIGURE 2:**
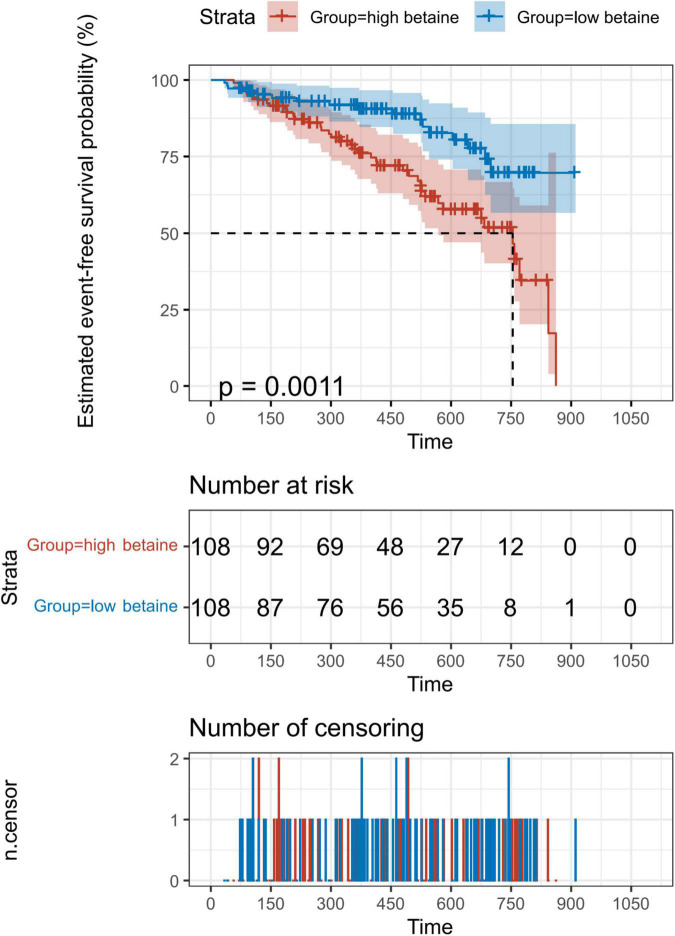
Kaplan–Meier (KM) analysis for the incidence of composite outcome events. Patients were divided into two groups according to the 50th percentile of plasma betaine concentrations (49.8 μM) at baseline. A total of 216 patients with PH were included for exploration. Value of *p* calculated by the log-rank test.

Kaplan–Meier analysis indicated the evidence that increased betaine level was associated with poor prognosis of patients with PH (Figure 2, *p* = 0.001). Univariate Cox regression analysis was performed, which is shown in Supplementary Table 3. After adjusting for confounders that include sex, WHO-FC, creatinine, blood urea nitrogen, and cardiac output index, multivariate Cox analysis revealed that high betaine was still correlated to poor clinical outcome among patients with PH [HR = 2.080, (95% CI: 1.033–4.188), *p* = 0.040, Table 2].

**TABLE 2:** Multivariate Cox analysis of plasma betaine levels and clinical outcomes.

Variable	HR	95% CI	*p*
Betaine (categorical variable)	2.080	1.033–4.188	0.040
Sex, female	0.801	0.354–1.812	0.595
WHO-FC	1.894	1.060–3.383	0.031
Creatinine, M m	1.027	1.002–1.052	0.037
BUN, Mm	0.845	0.685–1.042	0.116
Cardiac output index, L/(min/m^2^)	0.716	0.472–1.086	0.116

*Betaine represents plasma betaine concentrations. Plasma betaine levels were put into the model as a categorical variable bounded by 50th percentile (49.8 μM). WHO-FC, World Health Organization Function Class; BUN, blood urea nitrogen.*

### Betaine Was Responsive to Clinical Outcome

In this section, due to the undetectable betaine levels of blood samples collected in the second hospitalization, 13 patients with PH were excluded. During follow-up, patients received relative therapy, which was recommended by the 2015 European Society of Cardiology PH guideline (1). Here, Δbetaine and ΔNT-proBNP were calculated as the value at the second admission visit minus the baseline value.

**FIGURE 3:**
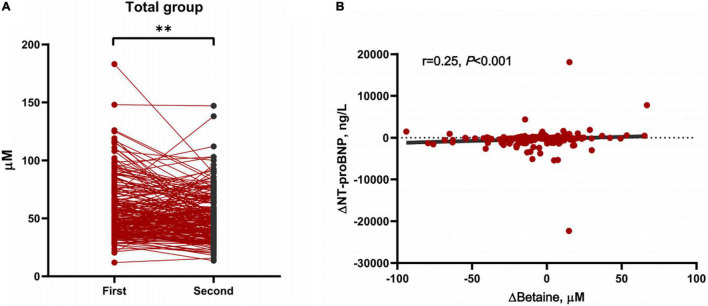
Changes of betaine between first and second hospitalization and the association between Δbetaine and ΔN-terminal pro-brain natriuretic peptide (NT-proBNP). **(A)** Demonstration of plasma levels of betaine after further guideline-recommended treatment in all PH participants (n = 203). Data were compared using paired-samples t-tests. **p < 0.001. **(B)** The positive association between Δbetaine and ΔNT-proBNP. Δbetaine and ΔNT-proBNP were defined as the value at the second admission visit minus the baseline value. Spearman’s correlation (2-tailed) was used for analysis.

**FIGURE 4:**
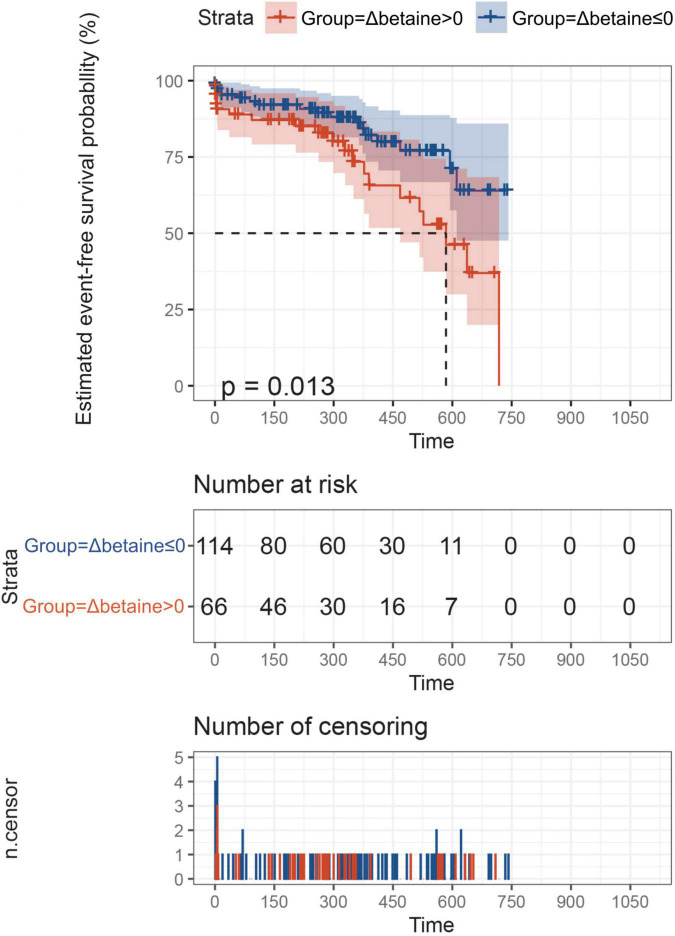
ΔBetaine was associated with the prognosis of patients with PH. Patients were divided into two groups according to Δbetaine >0 or Δbetaine ≤0. Follow-up duration was defined from the second betaine measurement to the occurrence of outcomes or the end of follow-up. In this analysis, outcome events occurred in 23 patients between two measurement points, so these patients were excluded. Finally, a total of 180 patients with PH were included for exploration. Value of *p* calculated by the log-rank test.

Betaine was decreased responsively among total patients [Δbetaine = −5.9 (−15.7, 7.3) μM, *p* < 0.001] positively with the decline of NT-proBNP (r = 0.25, *p* < 0.001, Figure 3). To further explore the association between Δbetaine and clinical outcomes, KM analysis was utilized and the result showed that Δbetaine >0 also indicated a poor prognosis of patients with PH (Figure 4).

## Discussion

Pulmonary hypertension is an under-recognized global health concern, and the general treatment of PH predominantly depends on the type and severity and the patient’s symptoms (14). Metabolomics provides the measurement of various metabolites in human samples, which holds great promise for the discovery of pathways linked to disease processes in clinical research (15). Researchers had observed associations between betaine with the prevalence of type 2 diabetes mellitus (16). Additionally, systemic levels of choline and betaine, two closely connected metabolites in the dietary lipid phosphatidylcholine, were also reported to be independently associated with the prevalence of CVDs (15). Moreover, a number of studies have reported increased concentration of betaine associated with the increased incidence of acute coronary syndromes and increased blood lipid concentrations (9, 17, 18). These studies suggest a potential link between betaine with type 2 diabetes mellitus and CVDs. However, less is known about the role of betaine in PH. In this study, we conducted a clinical study to assess the associations between plasma betaine concentration with severity and prognosis of patients with PH.

The current study’s 50th percentile of plasma betaine concentration was 49.8 mmol/L (interquartile range, 39.0–68.3), which was comparable with two cohorts of patients with CVDs (9, 19). Based on this baseline, we divided the study cohort into low and high betaine groups. Several key findings were noted in the baseline and correlation analysis. First, plasma betaine level was higher in older patients, and patients with high betaine were characterized by poor WHO-FC classification, higher NT-proBNP levels, larger RVD, and lower TAPSE and cardiac output index compared to those with low levels. Second, plasma betaine level was correlated positively with age, NT-proBNP, creatinine, and WHO-FC, and negatively with TAPSE, peak VO_2_, VO_2_%, and cardiac output index, of which WHO-FC, NT-proBNP, TAPSE, and cardiac output index remained statistically significant after adjusting for confounding factors. These correlating variables from the laboratory tests, echocardiography, and hemodynamics were all consensus-based criteria that have been identified to evaluate the severity and the risk stratification of patients with PH (1, 20). The worsening WHO-FC is not only a strong predictor of follow-up survival but also an alarming indicator of disease progression. NT-proBNP is not specific for PH but remains the biomarker in the routine practice to provide information of myocardial stress at the time of diagnosis and follow-up assessments, which is also regarded as a strong prognostic predictor in PH (21, 22). The cardiac output index assessed by RHC and the TAPSE measured by non-invasive echocardiogram are both robust reflections of heart function and are commonly used as indicators for severity assessment (1). Moreover, high levels of betaine remained robust to predict increased adverse prognosis regardless of potential confounders. Together, these results indicated that the plasma betaine level might have a positive correlation with PH severity in our study cohort. We had noticed no statistical differences in 6MWD and peakVO_2_ between groups were explored. This might attribute to the fact that severe patients with PH did not receive 6MWD and cardiopulmonary exercise tests, which narrowed the actual gap between the two groups.

To better understand the prognostic role of betaine in PH treatment, we conducted an analysis with follow-up ascertainment of primary endpoints in patients who received recommended therapies. Here, we found that both low plasma betaine and dynamic decreased levels were associated with a preferable prognosis in patients with PH. The results remind us of the role of NT-proBNP in PH and we reasonably postulate plasma betaine probably plays a similar role as a biomarker. Betaine changes could provide more information about the patient, and the lifestyle and diet of the patients included in the analysis are relatively consistent, which could largely reduce the effect of diet on betaine biosynthesis. In addition, Δbetaine was positively correlated with ΔNT-proBNP, indicating the dynamic change of betaine might reflect the disease condition and help for the management of PH.

The evidence showed that betaine enabled to protect internal organs, improve vascular risk factors, and enhance performance (8). The positive effects of betaine supplementation in treating hyper-homocystinurics (23, 24) and alcohol-induced hepatic steatosis (25) have also been elucidated. As a multifunctional agent, betaine possesses various physiological activities that include anti-oxidation, anti-inflammation, and anti-fibrosis (26–28). Notably, one study found that betaine exerted an anti-angiogenic activity ***in vivo*** and ***in vitro*** through the suppression of nuclear factor-κ B (NF-κ B) and Akt activation (29), another study showed betaine attenuated isoproterenol-induced acute myocardial ischemia *via* the regulation of signal transducer and activator of transcription 3 and apoptotic pathways (30). However, our study pointed out that high betaine levels were associated with poor clinical outcomes in patients with PH. Similarly, a meta-analysis also revealed that increased concentrations of betaine were correlated with the risk of major adverse cardiovascular events (31). We postulated that the increase of betaine in plasma might be a consequence due to disease progression rather than a cause. Lever et al. (19) speculated that betaine might be leaking from tissues where it was accumulated as an osmolyte. The leakage was a part of pathology in disease indicating the metabolic failure in patients. More severe patients probably have more leakage resulting in higher metabolite concentrations in plasma. However, further studies are needed to dissect the underlying mechanisms involved in this association.

Our study is by far the largest of its kind to examine the relationship of plasma betaine with PH development and prognosis. The cohort included PH patients without connective tissue disease, immune disease, acute coronary syndromes, active infection, malignancy, congestive heart failure, or diabetes. As such, our findings are less likely confounded by other pre-existing disease conditions and medication usage. However, several potential concerns or limitations are worth mentioning. Firstly, we do not have information regarding the dietary patterns, which are significantly related to the betaine concentration detected in the blood (32). Similarly, no information was available regarding non-prescriptive dietary supplements that may have been utilized by patients at the time of the study. Moreover, as a single-center cohort, this research was conducted on Chinese populations, where dietary habits differ dramatically from that of western countries. As such, the prospective association between plasma betaine and PH remains uncertain in western populations. Regardless, we would like to emphasize that our study findings are preliminary and were just hypothesize generating. Since this work was designed as an observational study, the associations in the current study cannot be considered causal, all findings need to be further investigated by large-scale randomized trials. Our findings, if further confirmed, call for collaborative research effort in the next decade could lead to significant further progress and perhaps even a cure of PH.

## Conclusion

The increase in plasma betaine was associated with PH severity and heralded a poor prognosis of patients with PH. Moreover, the dynamic rise of betaine level was also associated with adverse prognosis. Our study revealed the promising biomarker role of betaine and further explorations are calling for.

## Data Availability Statement

The raw data supporting the conclusions of this article will be made available by the authors, without undue reservation.

## Ethics Statement

The studies involving human participants were reviewed and approved by Ethics Committees of Fuwai Hospital (Approval No: 2018-1063). The patients/participants provided their written informed consent to participate in this study.

## Author Contributions

YY, JXu, JXue, and JZ contributed to the study design and interpretation of the results. YY, JXu, JXue, JZ, XL, BS, and BY contributed to the collection, analysis, or interpretation of data. YY and JXu prepared the manuscript. ZL, ZZ, QL, QZ, LZ, and CX critically revised the manuscript. All authors read and approved the final submitted version.

## Conflict of Interest

The authors declare that the research was conducted in the absence of any commercial or financial relationships that could be construed as a potential conflict of interest.

## Publisher’s Note

All claims expressed in this article are solely those of the authors and do not necessarily represent those of their affiliated organizations, or those of the publisher, the editors and the reviewers. Any product that may be evaluated in this article, or claim that may be made by its manufacturer, is not guaranteed or endorsed by the publisher.

## References

[1] Galiè N Humbert M Vachiery J Gibbs S Lang I Torbicki A. 2015 ESC/ERS Guidelines for the diagnosis and treatment of pulmonary hypertension: the joint task force for the diagnosis and treatment of pulmonary hypertension of the european society of cardiology (ESC) and the european respiratory society (ERS): endorsed by: association for european paediatric and congenital cardiology (AEPC), international society for heart and lung transplantation (ISHLT). *Eur Heart J.* (2016) 37:67–119. 10.1093/eurheartj/ehv317 26320113

[2] Tang W Wang Z Kennedy D Wu Y Buffa J Agatisa-Boyle B Gut microbiota-dependent trimethylamine N-oxide (TMAO) pathway contributes to both development of renal insufficiency and mortality risk in chronic kidney disease. *Circulat Res.* (2015) 116:448–55. 10.1161/circresaha.116.305360 25599331 PMC4312512

[3] Tang W Wang Z Levison B Koeth R Britt E Fu X Intestinal microbial metabolism of phosphatidylcholine and cardiovascular risk. *N Engl J Med.* (2013) 368:1575–84. 10.1056/NEJMoa1109400 23614584 PMC3701945

[4] Tang W Wang Z Shrestha K Borowski A Wu Y Troughton R Intestinal microbiota-dependent phosphatidylcholine metabolites, diastolic dysfunction, and adverse clinical outcomes in chronic systolic heart failure. *J Card Fail.* (2015) 21:91–6. 10.1016/j.cardfail.2014.11.006 25459686 PMC4312712

[5] Trøseid M Ueland T Hov J Svardal A Gregersen I Dahl C Microbiota-dependent metabolite trimethylamine-N-oxide is associated with disease severity and survival of patients with chronic heart failure. *J Int Med.* (2015) 277:717–26. 10.1111/joim.12328 25382824

[6] Koeth R Wang Z Levison B Buffa J Org E Sheehy B Intestinal microbiota metabolism of L-carnitine, a nutrient in red meat, promotes atherosclerosis. *Nat Med.* (2013) 19:576–85. 10.1038/nm.3145 23563705 PMC3650111

[7] Zeisel S Mar M Howe J Holden J. Concentrations of choline-containing compounds and betaine in common foods. *J Nutr.* (2003) 133:1302–7. 10.1093/jn/133.5.1302 12730414

[8] Craig S. Betaine in human nutrition. *Am J Clin Nutr.* (2004) 80:539–49. 10.1093/ajcn/80.3.539 15321791

[9] Wang Z Tang W Buffa J Fu X Britt E Koeth R Prognostic value of choline and betaine depends on intestinal microbiota-generated metabolite trimethylamine-N-oxide. *Eur Heart J.* (2014) 35:904–10. 10.1093/eurheartj/ehu002 24497336 PMC3977137

[10] Millard HR Musani SK Dibaba DT Talegawkar SA Taylor HA Tucker KL Dietary choline and betaine; associations with subclinical markers of cardiovascular disease risk and incidence of CVD, coronary heart disease and stroke: the Jackson Heart Study. *Eur J Nutr.* (2018) 57:51–60. 10.1007/s00394-016-1296-8 27550622 PMC5931705

[11] Tang W Wang Z Li X Fan Y Li D Wu Y Increased trimethylamine N-oxide portends high mortality risk independent of glycemic control in patients with type 2 diabetes mellitus. *Clin Chem.* (2017) 63:297–306. 10.1373/clinchem.2016.263640 27864387 PMC5659115

[12] Pulido T Adzerikho I Channick RN Delcroix M Galiè N Ghofrani HA Macitentan and morbidity and mortality in pulmonary arterial hypertension. *N Engl J Med.* (2013) 369:809–18. 10.1056/NEJMoa1213917 23984728

[13] Hoeper M Ghofrani H Grünig E Klose H Olschewski H Rosenkranz S. Pulmonary Hypertension. *Deutsch Arzteb Int.* (2017) 114:73–84. 10.3238/arztebl.2017.0073 28241922 PMC5331483

[14] Wang Z Klipfell E Bennett B Koeth R Levison B Dugar B Gut flora metabolism of phosphatidylcholine promotes cardiovascular disease. *Nature.* (2011) 472:57–63. 10.1038/nature09922 21475195 PMC3086762

[15] Lever M George P Slow S Bellamy D Young J Ho M Betaine and trimethylamine-N-oxide as predictors of cardiovascular outcomes show different patterns in diabetes mellitus: an observational study. *PLoS One.* (2014) 9:e114969. 10.1371/journal.pone.0114969 25493436 PMC4262445

[16] Danne O Lueders C Storm C Frei U Möckel M. Whole blood choline and plasma choline in acute coronary syndromes: prognostic and pathophysiological implications. *Clin Chim Acta* (2007) 383:103–9. 10.1016/j.cca.2007.05.001 17553478

[17] Lever M George P Atkinson W Molyneux S Elmslie J Slow S Plasma lipids and betaine are related in an acute coronary syndrome cohort. *PLoS One* (2011) 6:e21666. 10.1371/journal.pone.0021666 21747945 PMC3128609

[18] Lever M George P Elmslie J Atkinson W Slow S Molyneux S Betaine and secondary events in an acute coronary syndrome cohort. *PLoS One* (2012) 7:e37883. 10.1371/journal.pone.0037883 22649561 PMC3359285

[19] Gall H Felix J Schneck F Milger K Sommer N Voswinckel R The giessen pulmonary hypertension registry: survival in pulmonary hypertension subgroups. *J Heart Lung Transpl.* (2017) 36:957–67. 10.1016/j.healun.2017.02.016 28302503

[20] Leuchte H El Nounou M Tuerpe J Hartmann B Baumgartner R Vogeser M N-terminal pro-brain natriuretic peptide and renal insufficiency as predictors of mortality in pulmonary hypertension. *Chest.* (2007) 131:402–9. 10.1378/chest.06-1758 17296640

[21] Warwick G Thomas P Yates D. Biomarkers in pulmonary hypertension. *Eur Respir J.* (2008) 32:503–12. 10.1183/09031936.00160307 18669790

[22] Lawson-Yuen A Levy HL. The use of betaine in the treatment of elevated homocysteine. *Mol Genet Metab.* (2006) 88:201–7. 10.1016/j.ymgme.2006.02.004 16545978

[23] Truitt C Hoff WD Deole R. Health functionalities of betaine in patients with homocystinuria. *Front Nutr.* (2021) 8:690359. 10.3389/fnut.2021.690359 34568401 PMC8459993

[24] Rehman A Mehta KJ. Betaine in ameliorating alcohol-induced hepatic steatosis. *Eur J Nutr.* (2022). 61:1167–1176. 10.1007/s00394-021-02738-2 34817678 PMC8921017

[25] Bingül I Başaran-Küçükgergin C Aydın A Çoban J Doðan-Ekici I Doğru-Abbasoğlu S Betaine treatment decreased oxidative stress, inflammation, and stellate cell activation in rats with alcoholic liver fibrosis. *Environ Toxicol Pharmacol.* (2016) 45:170–8. 10.1016/j.etap.2016.05.033 27314760

[26] Ndisang J. The heme oxygenase system selectively modulates proteins implicated in metabolism, oxidative stress and inflammation in spontaneously hypertensive rats. *Curr Pharmaceut Design.* (2014) 20:1318–27. 10.2174/13816128113199990551 23978103

[27] Miwa M Tsuboi M Noguchi Y Enokishima A Nabeshima T Hiramatsu M. Effects of betaine on lipopolysaccharide-induced memory impairment in mice and the involvement of GABA transporter 2. *J Neuroinflamm.* (2011) 8:153. 10.1186/1742-2094-8-153 22053950 PMC3273450

[28] Yi E Kim Y. Betaine inhibits in vitro and in vivo angiogenesis through suppression of the NF-κB and Akt signaling pathways. *Int J Oncol.* (2012) 41:1879–85. 10.3892/ijo.2012.1616 22940742

[29] Zheng P Liu J Mai S Yuan Y Wang Y Dai G. Regulation of signal transducer and activator of transcription 3 and apoptotic pathways by betaine attenuates isoproterenol-induced acute myocardial injury in rats. *Hum Exp Toxicol.* (2015) 34:538–47. 10.1177/0960327114543936 25080425

[30] Heianza Y Ma W Manson JE Rexrode KM Qi L. Gut microbiota metabolites and risk of major adverse cardiovascular disease events and death: a systematic review and meta-analysis of prospective studies. *J Am Heart Associat.* (2017) 6:e004947. 10.1161/jaha.116.004947 28663251 PMC5586261

[31] Konstantinova S Tell G Vollset S Ulvik A Drevon C Ueland P. Dietary patterns, food groups, and nutrients as predictors of plasma choline and betaine in middle-aged and elderly men and women. *Am J Clin Nutr.* (2008) 88:1663–9. 10.3945/ajcn.2008.26531 19064529

