# Supplementary material for: High Betaine and Dynamic Increase of Betaine Levels Are Both Associated With Poor Prognosis of Patients With Pulmonary Hypertension
Source: Front Cardiovasc Med. 2022 Mar 30;9:852009. doi: 10.3389/fcvm.2022.852009 (PMC9005820; doi:10.3389/fcvm.2022.852009)
Supplement: Supplementary file 1 [file Table_1.docx]

**Supplementary Table 1. Correlation between betaine and clinical indicators**

| **Variables** | ***r*** | ***P* value** |
| --- | --- | --- |
| Age, years | 0.305 | **<0.001** |
| BMI, kg/m^2^ | 0.204 | **0.003** |
| **Laboratories** |  |  |
| NT-proBNP, pg/ml | 0.290 | **<0.001** |
| Albumin, g | -0.179 | **0.008** |
| Triglycerides | 0.009 | 0.895 |
| Total cholesterol, mM | -0.094 | 0.173 |
| Creatinine, μM | 0.354 | **<0.001** |
| **Echocardiography** |  |  |
| LVEF, % | -0.008 | 0.912 |
| RVD, mm | 0.124 | 0.073 |
| TAPSE, mm | -0.187 | **0.006** |
| **Hemodynamics** |  |  |
| mRAP, mmHg | 0.064 | 0.422 |
| mPAP, mmHg | -0.084 | 0.283 |
| cardiac output index, L/min*m^2^ | -0.304 | **<0.001** |
| PAWP, mmHg | 0.080 | 0.369 |
| PVR, WU | -0.019 | 0.825 |

Betaine represents plasma betaine concentrations. BMI: body mass index; NT-proBNP: N-terminal pro-brain natriuretic peptide; LVEF: left ventricular ejection fraction; RVD: right ventricular diameter; TAPSE: tricuspid annular plane systolic excursion; mRAP: mean right atrial pressure; mPAP: mean pulmonary atrial pressure; PAWP: pulmonary arterial wedge pressure; PVR: pulmonary vascular resistance.
