# Supplementary material for: High Betaine and Dynamic Increase of Betaine Levels Are Both Associated With Poor Prognosis of Patients With Pulmonary Hypertension
Source: Front Cardiovasc Med. 2022 Mar 30;9:852009. doi: 10.3389/fcvm.2022.852009 (PMC9005820; doi:10.3389/fcvm.2022.852009)
Supplement: Supplementary file 2 [file Table_2.docx]

**Supplementary Table 2. The associations between plasma betaine and WHO-FC, NT-proBNP, TAPSE, and cardiac output index after adjusting for confounders**

| **Logistics analysis** | **OR** | **95%CI** | **P** |
| --- | --- | --- | --- |
| **Model 1: WHO-FC** |  |  |  |
| Unadjusted | 2.205 | 1.260-3.859 | **0.006** |
| Adjusted ^a^ | 2.349 | 1.241-4.448 | **0.009** |
| **Model 2: NT-proBNP (categorical variable)** |  |  |  |
| Unadjusted | 2.273 | 1.303-3.966 | **0.004** |
| Adjusted ^b^ | 1.993 | 1.026-3.870 | **0.042** |
| **Model 3: TAPSE (categorical variable)** |  |  |  |
| Unadjusted | 1.904 | 1.103-3.286 | **0.021** |
| Adjusted ^b^ | 2.026 | 1.087-3.779 | **0.026** |
| **Model 4: cardiac output index (categorical variable)** |  |  |  |
| Unadjusted | 2.167 | 1.041-4.508 | **0.039** |
| Adjusted ^c^ | 2.390 | 1.087-5.255 | **0.030** |

Plasma betaine concentration was put into the model as a continuous variable.

The continuous variable of NT-proBNP, TAPSE, and cardiac output index were converted into a categorical variable with a boundary of 300pg/ml, 18mm, and 2.5L/min*m^2^.

^a^ Adjusted for age, sex, BMI, creatinine (continuous variable), and chronic kidney disease; ^b^ Adjusted for age, sex, BMI, creatinine(continuous variable), WHO-FC, and chronic kidney disease; ^c^ Adjusted for age, sex, BMI.

BMI: body mass index; WHO FC: world health organization function class; NT-proBNP: N-terminal pro-brain natriuretic peptide; TAPSE: tricuspid annular plane systolic excursion.
