# Supplementary material for: High Betaine and Dynamic Increase of Betaine Levels Are Both Associated With Poor Prognosis of Patients With Pulmonary Hypertension
Source: Front Cardiovasc Med. 2022 Mar 30;9:852009. doi: 10.3389/fcvm.2022.852009 (PMC9005820; doi:10.3389/fcvm.2022.852009)
Supplement: Supplementary file 3 [file Table_3.docx]

**Supplementary Table 3. Univariate Cox regression analysis between betaine and clinical indicators**

| Variable | **HR** | **95% CI** | ***P*** |
| --- | --- | --- | --- |
| Age, year | 1.013 | 0.997-1.030 | 0.111 |
| Sex, female | 0.585 | 0.345-0.992 | **0.046** |
| BMI, kg/m^2^ | 1.000 | 0.931-1.075 | 0.992 |
| WHO-FC | 1.550 | 1.066-2.254 | **0.022** |
| Betaine, μM (categorical variable) | 2.502 | 1.417-4.418 | **0.002** |
| NT-proBNP, pg/mL (categorical variable) | 2.231 | 1.215-4.095 | **0.010** |
| Creatinine, μM | 1.019 | 1.008-1.031 | **0.001** |
| BUN, mM | 1.095 | 1.000-1.198 | **0.049** |
| ALT, IU/L | 1.001 | 0.987-1.015 | 0.871 |
| AST, IU/L | 1.008 | 0.993-1.023 | 0.299 |
| Triglycerides, mM | 0.956 | 0.680-1.345 | 0.798 |
| LVEF, % | 0.968 | 0.926-1.012 | 0.148 |
| RVD, mm | 1.011 | 0.977-1.046 | 0.542 |
| TAPSE, mm | 0.951 | 0.883-1.025 | 0.186 |
| 6MWD,m | 1.001 | 0.998-1.004 | 0.553 |
| mRAP, mmHg | 1.064 | 0.992-1.141 | 0.083 |
| mPAP, mmHg | 0.994 | 0.976-1.012 | 0.501 |
| Cardiac output index, L/(min.m^2^) | 0.604 | 0.413-0.883 | **0.009** |
| PAWP, mmHg | 0.961 | 0.872-1.059 | 0.424 |
| PVR, wood | 0.993 | 0.931-1.060 | 0.840 |

Plasma betaine and NT-proBNP concentrations were converted to categorical variables with 49.8 μM and 300 pg/mL as the boundary, respectively.

BMI: body mass index; WHO FC: world health organization function class; NT-proBNP: N-terminal pro-brain natriuretic peptide; BUN: blood urea nitrogen; ALT: alanine aminotransferase; AST: aspartate aminotransferase; LVEF: left ventricular ejection fraction; RVD: right ventricular diameter; TAPSE: tricuspid annular plane systolic excursion; 6MWD: 6-minute walk distance; mRAP: mean right atrial pressure; mPAP: mean pulmonary atrial pressure; PAWP: pulmonary arterial wedge pressure; PVR: pulmonary vascular resistance.
