# Supplementary material for: High Betaine and Dynamic Increase of Betaine Levels Are Both Associated With Poor Prognosis of Patients With Pulmonary Hypertension
Source: Front Cardiovasc Med. 2022 Mar 30;9:852009. doi: 10.3389/fcvm.2022.852009 (PMC9005820; doi:10.3389/fcvm.2022.852009)
Supplement: Supplementary file 4 [file Image_1.pdf]

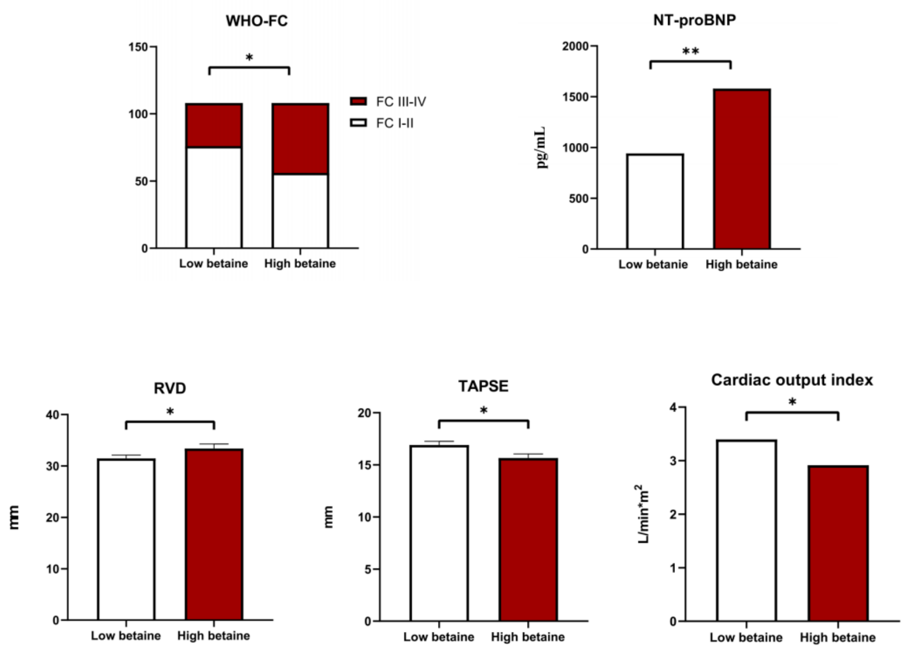

Supplementary Figure 1. Patients with elevated plasma levels of betaine received higher proportion of WHO-FC III-IV, higher NT-proBNP, larger RVD, lower TAPSE and cardiac output index compared to those with low betaine. PH patients (n=216) were stratified into low betaine and high betaine groups by 50th percentile of betaine (49.8 μM). WHO FC: world health organization function class; NT-proBNP: N-terminal pro-brain natriuretic peptide; RVD: right ventricular diameter; TAPSE: tricuspid annular plane systolic excursion. ***P***<0.05, ****P***<0.001
